# Aberrant Akt2 signaling in the RPE may contribute to retinal fibrosis process in diabetic retinopathy

**DOI:** 10.1038/s41420-023-01545-4

**Published:** 2023-07-13

**Authors:** Rachel Daley, Vishnu Maddipatla, Sayan Ghosh, Olivia Chowdhury, Stacey Hose, J. Samuel Zigler, Debasish Sinha, Haitao Liu

**Affiliations:** 1 Department of Ophthalmology, University of Pittsburgh School of Medicine, Pittsburgh, PA USA; 2 Wilmer Eye Institute, The Johns Hopkins University School of Medicine, Baltimore, MD USA

**Keywords:** Cell biology, Biotechnology

## Abstract

Diabetic Retinopathy (DR) is a complication of diabetes that causes blindness in adults. Retinal fibrosis is closely associated with developing proliferative diabetic retinopathy (PDR). Clinical studies have shown that fibrotic membranes exhibit uncontrolled growth in PDR and contribute to retinal detachment from RPE cells, ultimately leading to vision loss. While anti-VEGF agents and invasive laser treatments are the primary treatments for PDR, retinal fibrosis has received minimal attention as a potential target for therapeutic intervention. Therefore, to investigate the potential role of Akt2 in the diabetes-induced retinal fibrosis process, we generated RPE-specific Akt2 conditional knockout (cKO) mice and induced diabetes in these mice and *Akt2*^fl/fl^ control mice by intraperitoneal injection of streptozotocin. After an 8-month duration of diabetes (10 months of age), the mice were euthanized and expression of tight junction proteins, epithelial–mesenchymal transition (EMT), and fibrosis markers were examined in the RPE. Diabetes induction in the floxed control mice decreased levels of the RPE tight junction protein ZO-1 and adherens junction proteins occludin and E-cadherin; these decreases were rescued in *Akt2* cKO diabetic mice. Loss of Akt2 also inhibited diabetes-induced elevation of RNA and protein levels of the EMT markers Snail/Slug and Twist1 in the RPE as compared to *Akt2*^fl/fl^ diabetic mice. We also found that in *Akt2* cKO mice diabetes-induced increase of fibrosis markers, including collagen IV, Connective tissue growth factor (CTGF), fibronectin, and alpha-SMA was attenuated. Furthermore, we observed that high glucose-induced alterations in EMT and fibrosis markers in wild-type (WT) RPE explants were rescued in the presence of PI3K and ERK inhibitors, indicating diabetes-induced retinal fibrosis may be mediated via the PI3K/Akt2/ERK signaling, which could provide a novel target for DR therapy.

## Introduction

Diabetic retinopathy (DR) is one of the leading causes of blindness in adults of the modern world [1, 2]. In addition to the retinal vascular damage observed in early DR patients, retinal fibrosis has been closely associated with the development of proliferative DR (PDR), an advanced stage of DR, characterized by the presence of newly formed blood vessels often accompanied by epiretinal outgrowth of fibrotic membranes at the vitreoretinal interface [3, 4]. Retinal fibrosis is a process that typically occurs in response to hypoxia and inflammatory insults; clinical studies have shown that fibrotic membranes exhibit uncontrolled growth in PDR which contributes to retinal detachment from RPE cells, ultimately leading to vision loss [3, 4]. While anti-VEGF agents and invasive laser treatments serve as primary treatments for PDR at present, retinal fibrosis has received minimal attention as a target for potential therapeutic strategies. Therefore, further basic and clinical research is necessary to understand the role of retinal fibrosis in DR and for the development of potential therapeutics.

DR can be clinically classified into two stages: non-proliferative and proliferative [1]. Cell proliferation, extracellular matrix (ECM) expansion, and neovascularization are critical steps in progressing to PDR [5]. Increased production of ECM causes the basement membrane to thicken [6]. Hypoxia and inflammatory cytokines, characteristics of DR, have also been shown to promote ECM buildup and lead to the formation of fibrotic membranes on the retinal surface or in the vitreous cavity in DR [5, 7]. Many retinal cell types may be involved in this fibrotic process, including Müller cells, astrocytes, and microglia [7–10]. For instance, in diabetes, Müller cells are activated by chronic hyperglycemia. This results in increased cell proliferation and production of vascular endothelial growth factor (VEGF), which is a known to contributor of the retinal fibrosis process [11, 12]. The progression of fibrosis in diabetic retinopathy has been reported to be regulated by cyclin D and p27Kip1 - modulators of Müller cell activation [11]. Astrocytes are also cells of interest as they contribute to retinal fibrosis by secreting VEGF and fibronectin in mouse retina [13]. Further, in human diabetic retina microglia are reported to express Connective tissue growth factor (CTGF), which promotes ECM formation and the fibrosis processes [14]. In addition to Müller cells, astrocytes, and microglia, recent studies suggest that retinal pigment epithelial (RPE) cells may also contribute to retinal fibrosis in DR [3]. RPE cells, located between the neurosensory retina and the vascular choroid, constitute a monolayer of polarized multifunctional pigmented cells that form the outer blood-retina barrier (BRB) and play a crucial role in maintaining retinal function [15–17]. The breakdown of the outer blood retinal barrier observed in DR patients and diabetic mouse models [18] can activate RPE cells, initiating the epithelial–mesenchymal transition (EMT) process [19]. This leads to RPE cell proliferation, migration, and secretion of ECM molecules that contribute to retinal fibrosis and are involved in several diseases including PDR [19–21], age-related macular degeneration (AMD) [22], and proliferative vitreoretinopathy (PVR) [23].

It has been reported that high glucose can activate the PI3K/Akt signaling pathway in various cell types, including endothelial cells and podocytes [24, 25]. Such activation contributes to the expression of ECM in human renal proximal tubular cells [26]. Moreover, studies show that high glucose causes an increased expression of ECM molecules such as fibronectin, collagen IV, and laminin in RPE cells through the activation of PI3K/Akt signaling pathway, indicating PI3K/Akt in RPE could contribute to the formation of a fibrotic membrane during the development of DR [3]. However, the Akt isoform involved in this process needs to be clarified. A previous study has reported distinct roles of Akt1 and Akt2 in regulating cell migration and EMT in breast epithelial cells. Specifically, this study found that Akt1 down-regulation enhanced cell migration and EMT [27]. Interestingly, Akt2 down-regulation suppressed cell migration and the EMT process in IGF-IR–overexpressing cells [27]. These results highlight the distinct roles of Akt isoforms in EMT and cell migration that may be involved in the process of fibrosis in DR. Our previous study found that Akt1 and Akt2 activities were reciprocally regulated in the RPE of DR donor tissue and in diabetic mice, suggesting separate roles of the Akt isoforms within the RPE in DR [28]. While decreased Akt1 activity was found to contribute to retina vascular damage, the increased Akt2 activity in the RPE cells in the development of DR has not been explored. Here, we show that the RPE-specific knockout of *Akt2* attenuates diabetes-induced increases of EMT and fibrosis markers. Such beneficial effects may be due to activation of PI3K/Akt2/ERK signaling, which provides a foundation for targeting this signaling pathway as a therapeutic approach for treating retinal fibrosis in DR.

## Results

### Akt1 and Akt2 levels in non-diabetic and diabetic mouse and human RPE

**Fig. 1:**
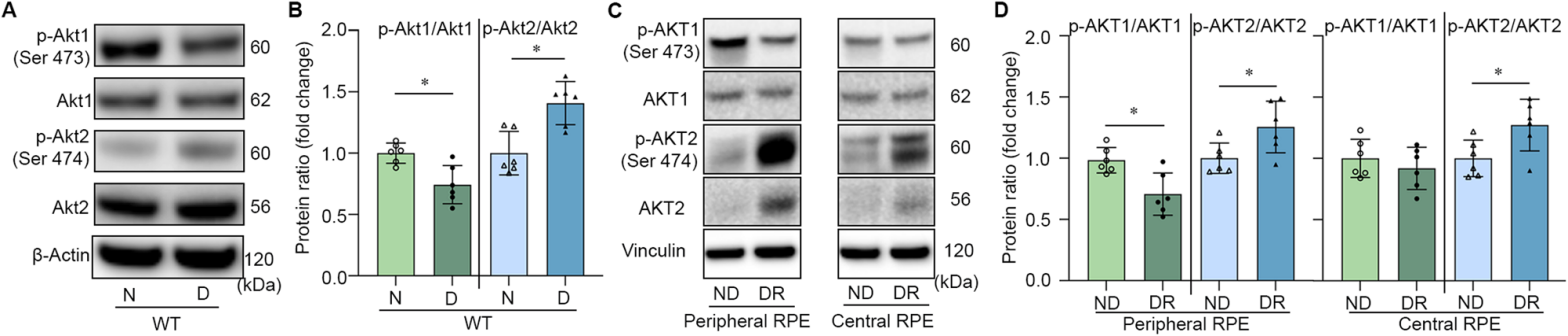
Effects of diabetes on Akt1 and Akt2 level in mouse and human RPE. Representative **A** immunoblots and **B** densitometry showing that diabetes decreased the p-Akt1/Akt1 ratio and increased the p-Akt2/Akt2 ratio in diabetic mouse RPE. Representative **C** immunoblots and **D** densitometry demonstrating that diabetes increased p-AKT2/AKT2 ratio in both peripheral and central RPE from human DR donors compare to non-diabetic controls. Diabetes decreased the p-AKT1/AKT1 ratio in peripheral RPE, but not in the central RPE in the same donor samples. In **A**–**D**, *n* = 6 for each group. Data are presented as mean ± SD. *p < 0.05. Statistical test used in this study is two tailed, unpaired *t*-test. N non-diabetic, D diabetic, ND non-diabetic, DR diabetic retinopathy.

To investigate the role of Akt in the retinal fibrogenesis in diabetes, we first examined the levels of phospho- and total Akt1 and Akt2 protein in RPE from diabetic mouse and human DR cadaver tissue. We found the protein ratio of phospho-Akt2/Akt2 to be highter in wild-type diabetic mouse RPE compared to non-diabetic mouse RPE, while the phospho-Akt1/Akt1 ratio was lower in wild-type diabetic mouse RPE compared to non-diabetic mouse RPE (Fig. 1A, B). This is consistent with what we previously reported [28]. In addition, alterations in AKT1 and AKT2 activity are also observed in the RPE of human DR donors. The ratio of phospho-AKT2/AKT2 is increased in both peripheral and central RPE collected from human DR donors compared to non-diabetic controls. However, the decreased ratio of phospho-AKT1/AKT1 was only observed in peripheral RPE (Fig. 1C, D), indicating region-specific changes in RPE protein levels. Basic characteristic of mouse and human RPE donors are provided in Supplementary Tables 1 and 2. Our previous study showed that the reduction in Akt1 activity in RPE contributes to the retinal vascular lesions in diabetic mice, but whether the increased Akt2 activity in DR RPE impacts disease pathology (progression) has not been investigated.

### Generation and validation of RPE-specific *Akt2* cKO mice

**Fig. 2:**
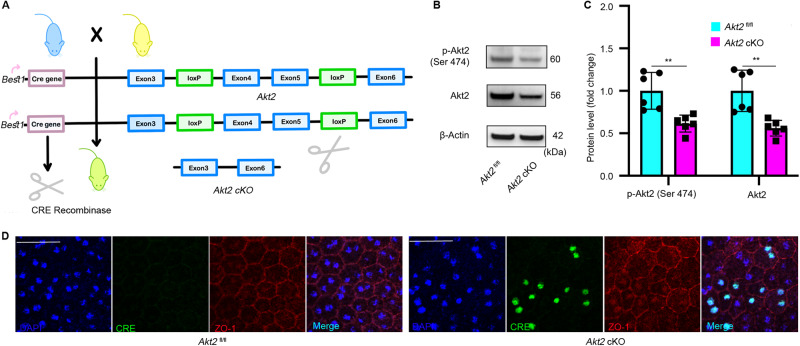
Generation of RPE-specific *Akt2* cKO mice. **A** RPE-specific *Akt2* cKO mice were generated by mating *Akt2*^fl/fl^ mice with *Best1*-Cre mice followed by cross-mating the progeny to generate the Cre-expressing mice homozygous for the floxed allele of *Akt2*. The *Akt2*^fl/fl^ mice contain loxP sites flanking exons 4 and 5 of Akt2; the *Best1*-Cre mice express Cre recombinase under the control of the RPE-specific *Best1* promoter. Thus, mating these two strains results in progeny where exons 4 and 5 of Akt2 are excised by Cre recombinase, making the resulting Akt2 protein dysfunctional, specifically in the RPE. **B** Representative immunoblot and **C** densitometry graph showing that the protein levels of p-Akt2 and total Akt2 are decreased in *Akt2* cKO RPE compared to *Akt2*^fl/fl^ RPE. **D** Representative RPE flat mounts stained for Cre (green) showing no Cre expression in *Akt2*^fl/fl^ mice and mosaic expression of the Cre in *Akt2* cKO mice. ZO-1, red; Scale bar: 50 µm. In **B**, *n* = 6. Data are presented as mean ± SD. **p < 0.01. Statistical test used in this study is two tailed, unpaired *t*-test. cKO conditional knock-out.

To elucidate the effects of increased Akt2 activity in RPE on the pathogenesis of DR, we generated RPE-specific *Akt2* cKO mice (Fig. 2A). To validate our *Akt2* cKO mouse model, we used immunoblotting and immunofluorescence assays. As expected, the protein levels of phospho-Akt2 and Akt2 were decreased in *Akt2* cKO RPE compared to *Akt2*^fl/fl^ RPE (Fig. 2B, C). RPE flat mounts stained for Cre (green) displayed mosaic expression of Cre in *Akt2* cKO mice (Fig. 2D) with no Cre expression in *Akt2*^fl/fl^ mice. Since the expression of Best1 is mosaic in the RPE cells, Akt2 expression is decreased but not completely absent in the cKO RPE. Both Akt1 and Akt2 have been reported to regulate EMT in various cell types including RPE and breast epithelial cells [19, 27]. High glucose and disrupted outer blood retinal barrier integrity [29] that is associated with DR has also been shown to induce EMT by contributing to the upregulation of fibrogenic factors in RPE cells [19]. Thus, we postulated that the increased Akt2 activity in RPE might contribute to EMT and retinal fibrosis in DR.

### *Akt2* cKO reduces diabetes-induced elevation of RNA levels of EMT markers in RPE

**Fig. 3:**
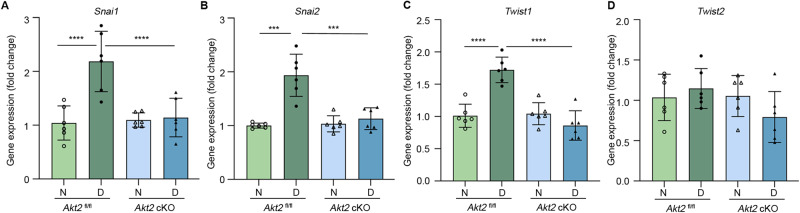
Effect of diabetes and *Akt2* cKO on the expression of EMT markers in RPE. 8 months of diabetes (10 months of age) significantly increased the mRNA levels of EMT markers *Snai1* (**A**), *Snai2* (**B**), and *Twist1* (**C**) but not for *Twist2* (**D**) in the RPE cells from *Akt2*^fl/fl^ diabetic mice; *Akt2* cKO inhibited this diabetes-induced increase. *n* = 6 mice for each group. Data are shown as mean ± SD. ***p < 0.001 and ****p < 0.0001. Statistical test used in this study is One-way ANOVA followed by Tukey’s post-hoc test. N non-diabetic, D diabetic, cKO conditional knock-out.

To investigate if EMT markers are associated with elevated Akt2 in the RPE of diabetic mice, we evaluated mRNA levels in the RPE of mice after an 8-month duration (10 months of age) of diabetes. The mRNA levels of EMT markers *Snai1*, *Snai2*, and *Twist1*, but not *Twist2*, were significantly increased in the RPE cells from *Akt2*^fl/fl^ diabetic mice (Fig. 3A–D). However, *Akt2* cKO diabetic mice did not display such alterations in EMT markers, suggesting that Akt2 might be involved in EMT in diabetic RPE.

### *Akt2* cKO attenuates the diabetes-induced increase of EMT marker proteins and decrease of tight junction proteins in RPE

**Fig. 4:**
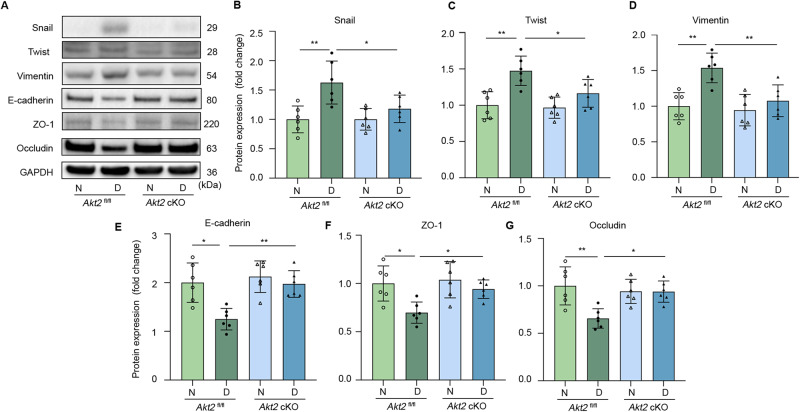
*Akt2* cKO attenuates diabetes-induced increased protein level of EMT markers and decreased tight junction proteins in RPE. **A** Representative RPE immunoblots and quantification of protein levels of EMT markers **B** Snail, **C** Twist1, **D** Vimentin, **E** adherens junction protein E-cadherin and **F**, **G** tight junction proteins ZO-1 and Occludin from 10 month old (8 months of diabetes) mice in *Akt2*^fl/fl^ and *Akt2* cKO RPE tissue. Diabetes significantly increased the levels of Snail, Twist1, and Vimentin, and decreased the levels of E-cadherin, ZO-1, and Occludin in the RPE cells from diabetic *Akt2*^fl/fl^ mice compared to non-diabetic controls. However, *Akt2* cKO significantly inhibited these diabetes-induced effects. *n* = 6 mice per group. Data are shown as Mean ± SD. *p < 0.05 and **p < 0.01. Statistical test used in this study is One-way ANOVA followed by Tukey’s post-hoc test. N non-diabetic, D diabetic, cKO conditional knock-out.

We further evaluated the protein levels of EMT markers in *Akt2*^fl/fl^ and *Akt2* cKO RPE tissue of the 10-month-old (8 months of diabetes) mice and found diabetes increased the protein levels of EMT markers Snail, Twist1, and Vimentin in that in control *Akt2*^fl/fl^ RPE, while *Akt2* cKO significantly inhibited the diabetes-induced changes in EMT markers (Fig. 4A–D), consistent with our mRNA data. Since a previous study found that disrupting tight junction proteins in RPE cells leads to the initiation of EMT [29], we examined the junctional proteins in our mouse model and found that loss of Akt2 in the RPE attenuated diabetes-induced decreases of the adherens junction protein E-cadherin and tight junction proteins ZO-1 and Occludin (Fig. 4E–G). This data indicates that disrupted junctional proteins might be involved the Akt2-regulated EMT process in diabetic RPE.

### Blocking Akt2 attenuates diabetes-induced RPE cell migration

**Fig. 5:**
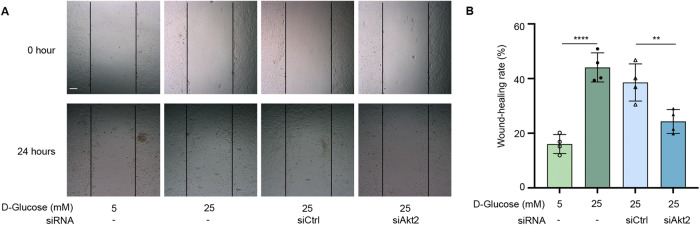
Blocking Akt2 attenuates diabetes-induced RPE cell migration. Human fetal RPE (fRPE) cells were transfected with and without non-relevant siRNA (siCtrl) or anti-Akt2 siRNA (siAkt2) for 24 h in 5 mM and 25 mM d-Glucose culture medium, and a wound healing experiment was performed. **A** Representative phase-contrast microscope images showing the area covered by the human fRPE cells at 0 and 24 h after wounding. **B** High glucose (25 mM) increased cell migration compared to the low glucose condition (5 mM), and blocking Akt2 signaling using Akt2 siRNA significantly inhibited diabetes-induced human fRPE cell migration compared to the control group (siCtrl). Cell migration was determined by the percentage of cells within the scratched area using ImageJ™ software. Data are collected from four replicates from each group and shown as mean ± SD. **p < 0.01 and ****p < 0.0001. Statistical test used in this study is One-way ANOVA followed by Tukey’s post-hoc test. Scale bar = 100 μm.

EMT is a process in which epithelial cells exhibit loss of apical-basal polarity with loosen cell–cell junctions to take on mesenchymal cell morphologies and invasive properties that facilitate migration through extracellular matrix. High glucose has been shown to promote the migration of RPE cells [19]. However, whether this process is mediated through Akt2 signaling is not clear. Our study confirmed that high glucose (25 mM) increases RPE cell migration compared to the low glucose condition (5 mM). Importantly, we found that blocking Akt2 signaling using Akt2 siRNA significantly inhibited high glucose-induced human fRPE cell migration compared to the control group (siCtrl), suggesting that Akt2 signaling contributes to the RPE cell migration/EMT process caused by diabetes (Fig. 5A, B). Since EMT in RPE cells is related to the pathogenesis of subretinal fibrosis [30], which is closely associated with the development of PDR and diabetic macular edema, we speculate that the diabetes-induced fibrosis process could be rescued in *Akt2* cKO mice.

### Attenuation of diabetes-induced RPE expression of fibrotic proteins through Akt2 deletion

**Fig. 6:**
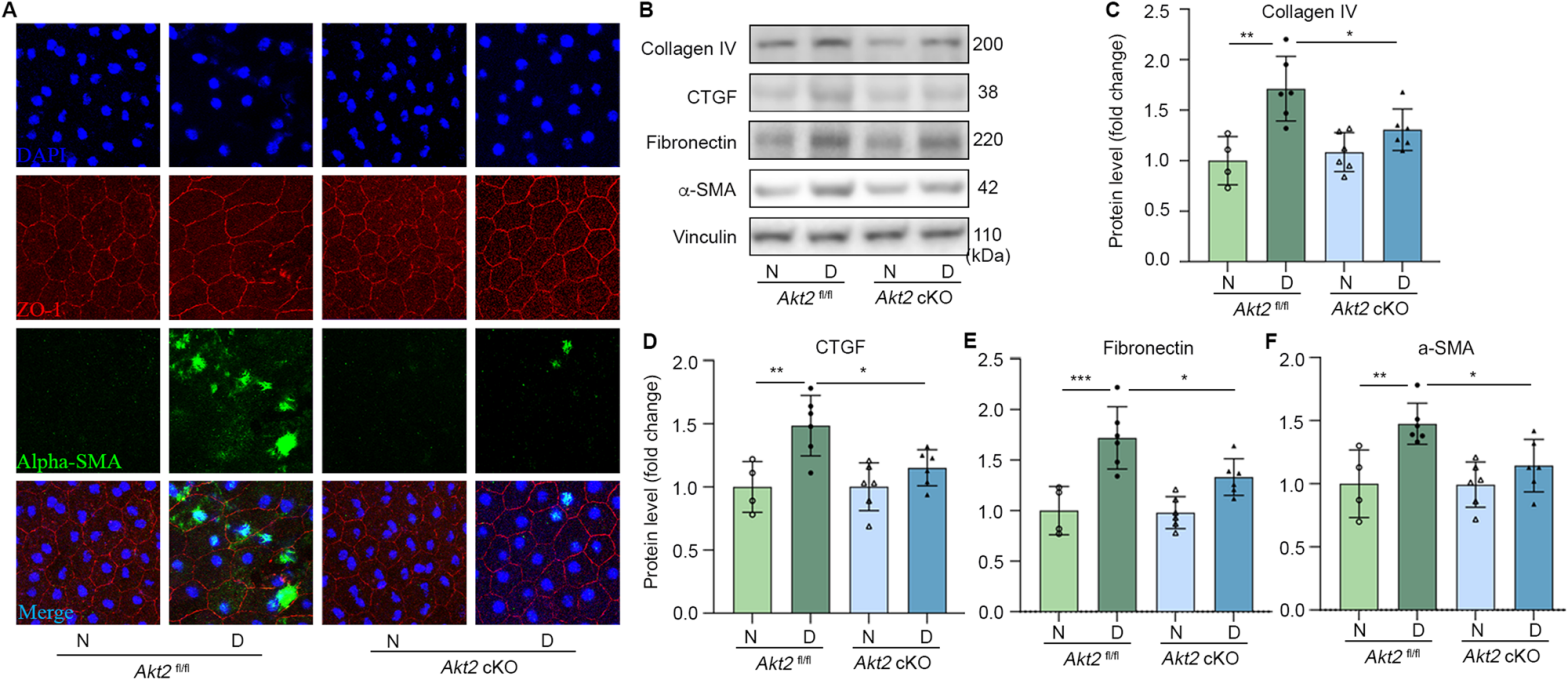
Fibrosis markers are decreased in RPE of diabetic mice with deletion of Akt2. **A** Representative RPE flat mounts stained for alpha-SMA (green) and ZO-1 (red) show that an 8 months duration of diabetes increased expression of the fibrosis marker alpha-SMA in diabetic *Akt2*^fl/fl^ mice, but not in *Akt2* cKO diabetic mice. DAPI, blue; merge, Cyan; Scale bar: 50 µm. **B** Representative immunoblots and densitometry showing that *Akt2* cKO inhibits a diabetes-induced increase in protein levels of Collagen IV (**C**), CTGF (**D**), Fibronectin (**E**), and alpha-SMA (**F**) in the RPE as compared to *Akt2*^fl/fl^ diabetic mice. In **B**–**F**, *n* = 4 mice for *Akt2*^fl/fl^ N, *n* = 6 for each of the other groups. Data are shown as Mean ± SD. *p < 0.05, **p < 0.01, and ***p < 0.001. Statistical test used in this study is One-way ANOVA followed by Tukey’s post-hoc test. N non-diabetic, D diabetic, cKO conditional knock-out.

To investigate if Akt2 contributes to the diabetes-induced retinal fibrosis process, we immunostained for the fibrosis marker alpha-SMA in RPE cells. After an 8 months diabetes induction, we found increased expression of alpha-SMA in RPE flat mounts from diabetic *Akt2*^fl/fl^ mice, but not in *Akt2* cKO diabetic mice (Fig. 6A). Immunoblotting confirmed this finding and further supported our hypothesis that knocking out Akt2 in RPE attenuates the diabetes-induced retinal fibrosis process by demonstrating that other fibrosis markers including collagen IV, CTGF, and fibronectin showed the similar pattern in the RPE (Fig. 6B–F). These data indicate that Akt2 signaling might contribute to the retinal fibrosis pathology in diabetes.

### Retinal fibrosis process induced by diabetes may be mediated through PI3K/Akt2/ERK signaling

**Fig. 7:**
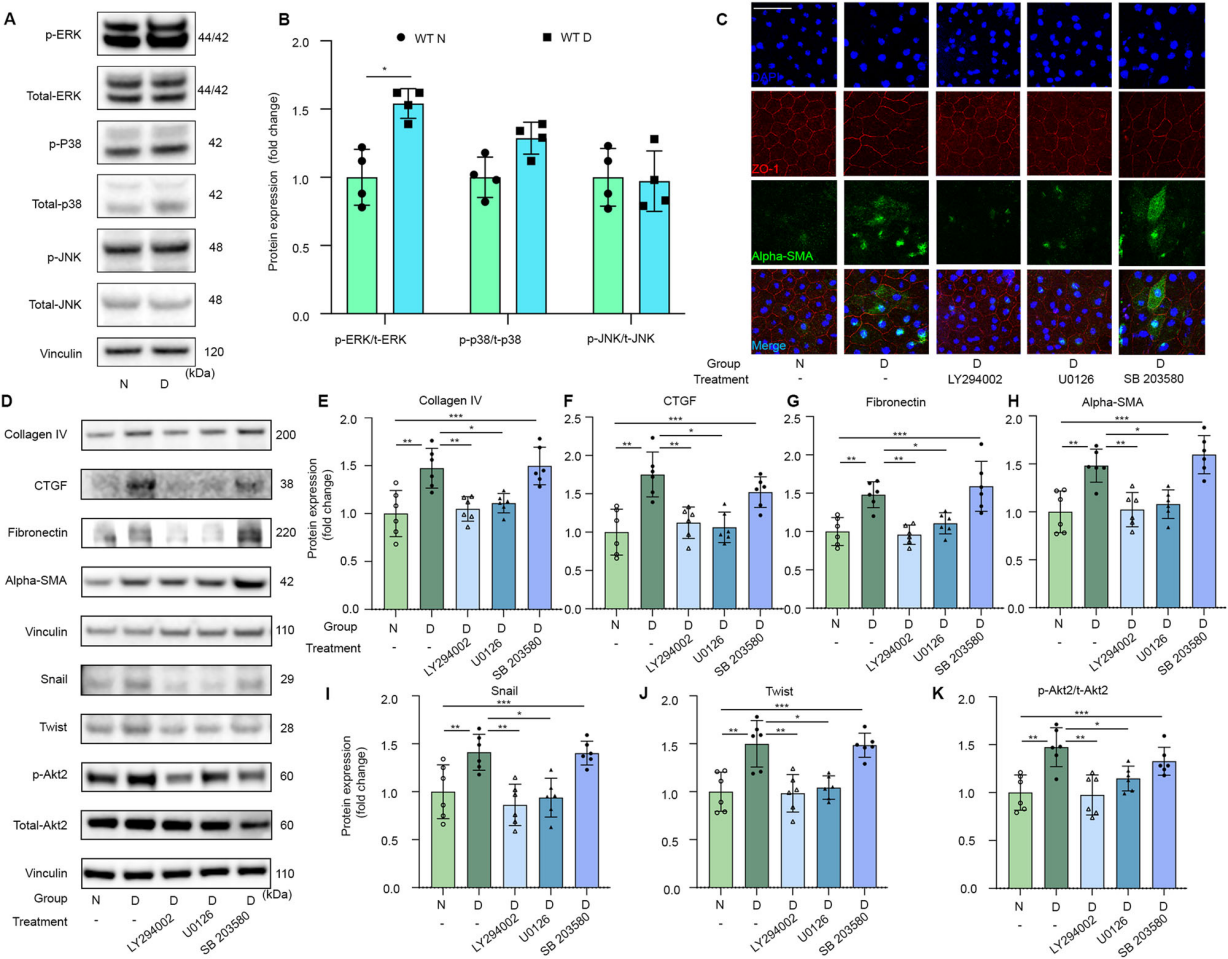
Diabetes-induced increased of retinal fibrosis markers in RPE are inhibited in the presence of PI3K and ERK inhibitors. Representative immunoblot (**A**) and densitometry graph (**B**) showing that diabetes increased the p-ERK/t-ERK ratio in RPE cells. *n* = 4 mice for each group. Data are shown as Mean ± SD. Two tailed, unpaired *t*-test was used to analyze the data, *p < 0.05. **C** Representative RPE flat mounts stained for alpha-SMA (green) and ZO-1 (red) show normal glucose condition or high glucose treated with PI3K and ERK inhibitor, but not for P38 inhibitors, display less staining of alpha-SMA when compared to the untreated high glucose group. Representative immunoblot (**D**) and densitometry graph further confirmed that diabetes-induced increases of fibrosis markers including collagen IV (**E**), CTGF (**F**), fibronectin (**G**), and alpha-SMA (**H**) and EMT markers such as snail (**I**) and twist (**J**) were all attenuated in the presence of PI3K and ERK inhibitors, but not with the p38 inhibitor. The PI3K and ERK inhibitors both also reduced Akt2 activity in RPE. In **D**–**K** data are shown as mean ± SD, *n* = 6 mice for each group. *p < 0.05, **p < 0.01, and ***p < 0.001. Statistical test used in this study is One-way ANOVA followed by Tukey’s post-hoc test. N non-diabetic, D diabetic.

To further explore the underlying mechanism whereby Akt2 regulates EMT and the fibrosis process in the diabetic retina, we investigated the MAPK signaling. Previous studies have shown that MAPK signaling is activated in diabetes, and MAPK signaling contributes to the pathogenesis of fibrosis in eye disease [31, 32]. Our previous research work has shown that the increased Akt2 activity is due to the ROS-induced activation of PI3K/PKD1 signaling [28]. Thus, we postulate that diabetes-induced EMT and fibrosis in diabetic mice RPE cells is due to PI3K/Akt2/MAPK signaling. To investigate this idea, we performed an immunoblotting study and found the ratio of phospho-ERK/total ERK to be higher in the RPE tissue from diabetic mice compared to non-diabetic controls. In contrast, the ratio of phospho-p38/total p38 showed higher trend in the RPE of diabetic than non-diabetic mice, but the difference was not statistically significant (Fig. 7A, B). There was also no significant difference in the phospho-JNK/total-JNK ratio of the diabetic and non-diabetic group (Fig. 7A, B). Thus, we postulate that Akt2-regulated EMT and fibrosis processes might be regulated through Akt2/ERK signaling.

To further test this hypothesis, RPE flat mounts from non-diabetic and diabetic mice were cultured in low and high glucose conditions with and without PI3K inhibitor (LY294002), ERK inhibitor (U0126), and P38 inhibitor (SB 203580). Immunostaining demonstrated that RPE flat mounts exposed to high glucose conditions and treated with PI3K and ERK inhibitors displayed significantly less intensity for alpha-SMA compared to the high glucose group not treated with any inhibitors (Fig. 7C). Immunoblotting confirmed this result and showed that diabetes-induced elevation of other fibrosis markers including collagen IV, CTGF, and fibronectin, and EMT markers such as Snail and Twist were attenuated in the presence of PI3K and ERK inhibitors, but not with the p38 inhibitor (Fig. 7D–J). Also, the PI3K inhibitor significantly reduced Akt2 activity in RPE (Fig. 7K), consistent with our previous finding. Moreover, the ERK inhibitor inhibits Akt2 activity in RPE in diabetic conditions (Fig. 7K), indicating that Akt2 and ERK may mutually regulate each other. Data shows that PI3K/Akt2/ERK signaling contributes to the diabetes-induced retinal fibrosis process. In summary, deletion of Akt2 in the RPE inhibits the diabetes-induced elevation of EMT and fibrosis markers, and prevents the diabetes-induced alteration of the tight junction proteins. Furthermore, blocking PI3K or ERK signaling has a similar beneficial effect in inhibiting high glucose-induced EMT and fibrosis markers. Thus, the PI3K/Akt2/ERK signaling pathway in RPE cells might be involved in retinal fibrosis pathogenesis in DR, contesting this signaling pathway as a potential therapeutic target to delay the progression of DR.

## Discussion

DR is a common complication of diabetes mellitus and is one of the leading causes of visual impairment and blindness in middle-aged and elderly individuals [33]. If untreated, DR progresses from a mild, non-proliferative stage to a moderate, and severe stage, and finally to PDR where retinal fibrosis occurs with increased ECM deposition and formation of fibrotic membranes, which in turn lead to retinal detachment and vision loss [3, 4]. Anti-VEGF agents and invasive laser treatments are current therapies available for PDR patients, however, potential therapeutic strategies targeting the retinal fibrosis process remain largely unexplored. Here, we explored potential mechanisms underlying the retinal fibrosis process in DR to facilitate the development of targeted interventions for treating retinal fibrosis in DR.

While studies focusing on the distinct roles of the Akt1 and Akt2 isoforms in RPE during the development of DR have not been published, the idea that Akt may be involved in RPE cell EMT and retinal fibrosis in diabetes has recently surfaced. Akt1 and Akt2 are functionally distinct isoforms. It has been reported that Akt2 whole-body knockout mice display a severe type-II diabetes phenotype and that human patients with mutations in Akt2 show severe insulin resistance and develop diabetes [34, 35]. In the RPE, Akt2 is activated in both DR cadaver tissues and diabetic mice, implicating Akt2 in DR pathogenesis [36, 37]. Further, a previous study reported that Akt1 down-regulation enhanced cell migration and EMT, while Akt2 down-regulation suppressed cell migration and EMT in IGF-IR–overexpressing cells [27]. These results highlight the distinct roles of Akt isoforms in EMT and cell migration, processes involved in the fibrotic changes occurring in DR. These studies indicate Akt2 could be a key regulator in the retinal fibrotic process in DR. Here, we report for the first time that in RPE cells from both human DR eyes and diabetic mice, Akt2 activity is increased and that this increase is associated with EMT and cell migration. Furthermore, we show that in an Akt2 knockout mouse, specifically in the RPE, the diabetes-induced increases of RPE EMT markers in vivo and cell migration in vitro were significantly lowered, indicating that Akt2 signaling in RPE cells might contribute to the RPE EMT in DR. In addition, decreased ZO-1 expression as observed in diabetic RPE cells was restored in the *Akt2* cKO mice. Disruption of ZO-1 enhanced RPE cells for migration and triggered the EMT process, suggesting that breakdown of the outer retinal blood barrier might drive the RPE cell migration and activate the EMT process through Akt2 signaling.

CTGF is a fibrogenic factor that has been implicated in DR [38]. A previous study has shown CTGF inhibitor, SERPINA3K, reduces retinal ECM production and fibrogenic activity in diabetic rat retina [38]. It also showed that CTGF-siRNA decreased retinal ECM components and collagen IVα3 mRNA levels in diabetic rats [39]. In another study, TNF-alpha treatment increased mRNA levels of collagen IV (component of ECM) and Fibronectin1 in high glucose cultured hiPSC-RPEs compared to normal glucose controls, indicating inflammatory cytokines contribute to fibrogenesis in DR [40, 41]. Moreover, when RPE cells undergo EMT at high glucose conditions, they lost polarity and began expressing alpha SMA [19, 42], another important marker of the fibrosis process. Here, we demonstrate that knocking out Akt2, specifically in the RPE, attenuated the diabetes-induced increase in fibrotic markers including CTGF, Collagen IV, Fibronectin, and alpha-SMA, suggesting the involvement of Akt2 in the fibrosis process of DR.

**Fig. 8:**
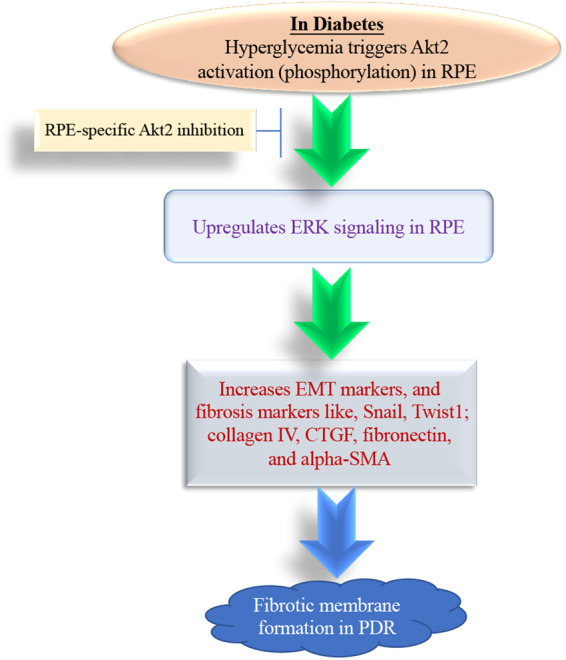
A schematic illustration of abnormal Akt2 signaling in the RPE may contribute to retinal fibrosis in PDR. Hyperglycemia triggers Akt2 activation (phosphorylation) in the RPE and increases the expression of EMT markers and fibrosis proteins through ERK signaling, these alterations are inhibited in RPE-specific *Akt2* cKO diabetic mice, indicating Akt2 signaling in RPE may contribute to the retinal fibrotic membrane formation in PDR. EMT epithelial–mesenchymal transition, PDR proliferative diabetic retinopathy.

Emerging evidence show that Akt activity triggered by ROS leads to the activation of MAPK [43], which can regulate EMT and cell migration, both of which are heavily involved in the retinal fibrosis process in DR. In this study, we investigated the mechanism underlying the fibrosis process regulated by Akt2. We found that elevated Akt2 activity in the RPE of diabetic mice is associated with increased ERK activity concomitant with increased expression of EMT and fibrosis markers. Additionally, ERK and PI3K inhibitors reduced the expression of fibrosis markers in RPE induced by hyperglycemia (Fig. 8). ROS has been shown to stimulate the PI3K signaling and activate Akt2 in RPE under diabetic conditions, suggesting that retinal fibrosis in diabetes might be attributed to the increased ROS in the eye activating Akt2/ERK signaling.

Inflammation plays a detrimental role in the progression of DR [2], and has been shown to contribute to retinal fibrosis. Our previous study show that Akt1 in RPE regulate inflammation [44]. In this case, decreased RPE Akt1 activity upregulate inflammatory cytokines in diabetic retina which might in turn contribute to retinal fibrosis. However, minimal the effect, as shown in our previous study, decrease in Akt1 activities is mainly involved in retinal capillary damages in diabetic mice. Instead of Akt1, here we show increased Akt2 activity augments the retinal fibrosis process. Collectively, our results demonstrate complex Akt2 signaling in the RPE during the progression of DR and identify a pathway (Akt2/ERK) that could be manipulated to develop new therapies for the treatment of diabetic eye disease.

## Materials and methods

### Animals

Wild-type C57BL/6J mice were purchased from the Jackson Laboratory (Bar Harbor, ME, USA, 000664). RPE-specific *Akt2* cKO mice were generated by mating the *Akt2*^fl/fl^ mice (The Jackson Laboratory, Bar Harbor, ME, USA, 026475) with *Best1/Cre* mice (The Jackson Laboratory, Bar Harbor, ME, USA, 017557) followed by cross-mating the progeny to generate the Cre-expressing mice homozygous for the *Akt2*^fl/fl^. The *Akt2*^fl/fl^ mice possess loxP sites flanking exons 4 and 5 of *Akt2*; the *Best1/Cre* mice express Cre recombinase under the control of Best1 promoter. Thus, mating these two strains results in the progeny, where exons 4 and 5 of *Akt2* are excised by Cre recombinase controlled by the Best1 promoter, making the resulting Akt2 protein dysfunctional only in the RPE. All animals were housed in ventilated microisolator cages under 12/12 hours of light and dark cycle. Animal procedures followed the NIH Guide for the Care and Use of Laboratory Animals, and were authorized by the University of Pittsburgh’s Institutional Animal Care and Use Committee.

### Genotyping

Tail snips were collected from 2–4 week old *Akt2* cKO mice. DNA extraction was done by using a DNeasy Blood and Tissue kit (Qiagen, Germantown, MD, USA, 69506). The purified DNA was mixed with 1-Drop PCR master Mix (with dye) (101 Bio, Mountain View, CA, USA, W2599-5), and optimized Cre forward and reverse primers: F (ATG CCC AAG AAG AGG AAG GTG TC) R: (TGG CCC AAA TGT TGC TGG ATA GTT TTT A). The PCR reaction was then amplified using an Applied Biosystems MiniAmp Thermal Cycler with optimized PCR reaction conditions. The amplified DNA was then run on a 1–2% ethidium bromide (Invitrogen, Carlsbad, CA, USA, 15585-011) agarose gel (Sigma-Aldrich, St. Louis, MO, USA, A9539-500G) at 120 volts for 30 min. The gel was imaged on a Biosystems Azure 400 fluorescent imager analyzing the molecular weight compared to TrackIt &Trade 100 bp ladder (Invitrogen, Carlsbad, CA, USA, 10488058).

### Induction of diabetes

Animals were randomly allocated into experimental groups using Microsoft Excel-generated randomization method, and mice around 2 months of age were intraperitoneally injected with streptozotocin (STZ, Sigma Aldrich, St. Louis, MO, S0130) and resuspended in citrate buffer at a concentration of 60 mg/kg for five consecutive days [45]. Fasted blood glucose (FBG, fasted for 6 hours) was measured between 7 and 15 days after the last STZ injection. the onset of diabetes is defined when FBG readings exceed 275 mg/dL on three different days. To assess the severity of hyperglycemia or diabetic status, Hemoglobin A1c (HbA1c) was measured using Mouse HbA1c Assay Kit (Crystal Chem, Elk Grove Village, IL, USA, 80310) and its relative control (Crystal Chem, 80313). In addition, we monitored mouse body weight 2–3 weeks after the injection of STZ. Subcutaneous injection of insulin (0–0.2 units, Invitrogen, 12585014) were administered as needed to prevent weight loss while still allowing hyperglycemia (Supplementary Table 1).

### Western blotting

RPE and retina lysates were prepared from RPE-choroid, RPE cell lines, and retina. Samples were sonicated in RIPA lysis buffer (EMD Millipore, Burlington, MA, 20-188) with 1% phosphatase (Sigma-Aldrich, P0044) and protease inhibitors (Sigma-Aldrich, I3786), then centrifuged at 13,000 g for 20 min at 4 °C. The supernatants were collected, and protein concentrations were estimated by a BCA kit (Thermo Fisher Scientific, Waltham, MA, 23225). Samples were adjusted to the same concentration by adding lysis buffer, then mixed with 4X protein sample buffer (Life Technologies, Carlsbad, CA, NP0007) with 5% 2-mercaptoethanol (Sigma Aldrich, M3148) and heated at 96 °C for 10 min. Total protein (10 μg for RPE, 20ug for retina) was loaded onto 4–12% Bis-Tris Nu-PAGE gels (Thermo Fisher Scientific, NW04125BOX), proteins were transferred onto nitrocellulose membranes after electrophoreses were performed in MES buffer (Thermo Fisher Scientific, B0002), and the membrane was blocked with 5% milk (Bio-Rad, Hercules, CA, 170-6404) for an hour, followed by primary antibodies at a dilution of 1:1000 overnight. Primary antibodies are as follows: Phospho-Akt2 (Ser474) (Cell Signaling Technology, Danvers, MA, 8599S), Akt2 (Cell Signaling Technology, 3063S), Phospho-Akt1 (Ser473), (Cell Signaling Technology, 9018S), Akt1 (Cell Signaling Technology, 2938S), Snail (Thermo Fisher Scientific, 14-9859-82), Twist1 (Thermo Fisher Scientific, PA5-49688), Vimentin (Proteintech, Rosemont, IL, 10266-1-AP), E-cadherin (Cell Signaling Technology, 3195S), ZO-1 (Thermo Fisher Scientific, 402200), GAPDH (Cell Signaling Technology, 5174S), Vinculin (Abcam, Boston, MA, ab129002), CTGF (Santa Cruz Biotechnology, Santa Cruz, CA, sc-14939, Clone L-20), Fibronectin (Sigma Aldrich, AB2033), Collagen IV(Sigma Aldrich, AB769), α-Smooth Muscle Actin (alpha-SMA, Cell Signaling Technology, 19245, Clone D4K9N), and Occludin (Thermo Fisher Scientific, 91131S). The membranes were washed three times with TBS (Thermo Fisher Scientific, 351-086-131) with 0.1% Tween (Sigma Aldrich, P7949) for 10 min per wash and incubated with appropriate peroxidase-labeled goat anti-rabbit secondary antibody (SeraCare, Gaithersburg, MD, 5220-0336) at a dilution of 1:2000 for 1 h at room temperature, followed by another three washes in TBS-Tween. The membranes were then developed using ECL Western Blotting Detection Reagent (GE Healthcare, Chicago, IL, RPN2209) and the Azure c400 system [46].

### Immunostaining

The mice were anesthetized by CO_2_ asphyxiation, freshly dissected whole eyes were fixed in 4% paraformaldehyde (PFA) for 2 h and then the anterior parts and retina were removed. RPE-choroid flatmounts were first permeabilized with 0.25% Triton X-100 in PBS for 10 min, followed by 2% donkey serum, 2% goat serum, 1% BSA, and 0.1% Triton X-100 in PBS (blocking buffer) for 30 min at room temperature. *Akt2*^fl/fl^and *Akt2* cKO RPE flatmounts were incubated with Cre antibody (1:200, Sigma-Aldrich, MAB3120, Clone: 2D8) or alpha-SMA, (1:200, Cell Signaling Technology, 19245, Clone D4K9N) in blocking buffer at 4 °C overnight. RPE flatmounts were washed in 1X PBS for 3 times, 5 min per wash. The secondary antibody Donkey anti-Rabbit, Alexa Fluor 488 (1:200, Invitrogen, A21206), Donkey anti-mouse, Alexa Fluor 488 (1:200, Invitrogen, A21202), 1 μg/mL DAPI (1:400, Thermo Fisher Scientific, D1306), and Alexa Fluor 594 conjugated ZO-1 (1:200, Invitrogen, 339194) antibody were applied to appropriate samples for 1 h incubation at room temperature. RPE flatmounts were cover slipped with DAKO mounting medium (Agilent, Santa Clara, CA, S3023). Images were acquired by a Zeiss LSM 710 confocal workstation.

### siRNA transfection

The human fetal RPE cells derived from healthy human RPE cells, was a gift from Dr. Ram Kannan, Macular Research Lab. Informed consent was obtained from the patients for their use. The human fetal RPE (fRPE) cells retain many in vivo phenotypic characteristics. RPE-specific markers such as RPE65 and epithelial markers ZO-1 are expressed in the human fetal RPE cells. The cells were seeded and incubate in six-well culture plate at low (5 mM) and high glucose (25 mM) for 2 days. Cells were then transfected with signal silence control siRNA (Cell Signaling Technology, 6568) and *Akt2* siRNA (Cell Signaling Technology, 6407) using Lipofectamine 3000 (Thermo Fisher Scientific, L3000008) following the manufacturer’s protocol. The silencing efficiency was detected by western blotting 48 h after transfection.

### RNA isolation and real-time RT-PCR

Total RNA was isolated, and cDNA was generated as described previously [47]. Briefly, RPE-choroid was isolated and placed into a 1.5 ml Eppendorf tube with 200 μl RNA Protect Cell Reagent (Qiagen, 76526) for 10 min at room temperature. The tube was briefly agitated to ensure most of the RPE cells were released, and then the eyecup was removed. Samples were centrifuged at 2500 rpm (685 × *g*) for 5 min to pellet the RPE cells, discarding the supernatant and the RNA isolation was performed using Isolate II RNA Mini kit (Bioline, Memphis, TN, BIO-52072), according to the manufacturer’s instructions. cDNA was generated using SuperScript® VILO™ cDNA Synthesis Kit (Invitrogen, 11754-050). The mRNA expression of mouse Snai1 (Thermo Fisher Scientific, Mm00441533_g1), Snai2 (Thermo Fisher Scientific, Mm00441531_m1), Twist1 (Thermo Fisher Scientific, Mm04208233_g1), Twist2 (Thermo Fisher Scientific, Mm00492147_m1) and β-actin (Thermo Fisher Scientific, Mm00607939_s1) genes were evaluated by using QuantStudio 3 qPCR machine (Applied Biosystems by Thermo Fisher Scientific, A28131) after samples were mixed with Taqman probes and TaqMan Gene Expression Master Mix (Thermo Fisher Scientific, 4369514). The gene expression levels were normalized relative to β-actin mRNA and reported as fold change over controls using the Delta delta Ct method.

### Wound healing assay

For the wound healing assay, human fPRE cells were seeded into six-well culture plate. Cells were seeded at 4 × 10^5^ per well of a 6-well plate cultivated for 48 h to achieve complete spreading of cells and 100% of monolayer confluency. The monolayer wounding was performed after cells were transfected with Akt2 siRNA as mentioned in siRNA transfection section for 24 h. Gentle cross scratching with a 200 μl tip or polished toothpick sterilized with ethanol was applied to every well. Images were acquired by microscopic imaging.

### Statistical analysis

Experiments are conducted blinded and randomized. We performed our study with numbers usually included in this kind of analysis [28]. The p-values are determined by either One-way ANOVA followed by Tukey’s post-hoc test (multiple-group comparisons), or a two-tailed unpaired Student’s t-test (comparison between two groups). For the invitro study, the experiment was performed in triplicate, and the presented data is from one representative experiment out of at least three independent experiments that yielded consistent results. Significance is defined as *p < 0.05, **p < 0.01, ***p < 0.001, ****p < 0.001. Statistical analysis was performed using GraphPad 6.0 software (GraphPad Software, Inc., La Jolla, CA, USA); All data were expressed as mean ± SD.

## Supplementary information

Cover Sheet for Supplemental Data files

Supplementary Table

Supplementary Table Legends

Original Data File

## Data Availability

All data generated in this study are included in this published article and its supplementary information files.
