# Supplementary material for: Aberrant Akt2 signaling in the RPE may contribute to retinal fibrosis process in diabetic retinopathy
Source: Cell Death Discov. 2023 Jul 13;9:243. doi: 10.1038/s41420-023-01545-4 (PMC10345150; doi:10.1038/s41420-023-01545-4)
Supplement: Supplementary file 1 — Cover Sheet for Supplemental Data files [file 41420_2023_1545_MOESM1_ESM.docx]

Supporting Information for

**Aberrant Akt2 signaling in the RPE may contribute to the retinal fibrosis process in Diabetic Retinopathy**

Rachel Daley et al.

Corresponding author: Haitao Liu, [hal140@pitt.edu](mailto:hal140@pitt.edu); Debasish Sinha, [Debasish@pitt.edu](mailto:Debasish@pitt.edu);

This PDF file includes:

Supplementary Tables 1 – 2, and Supplementary File

1- File Name: **Supplemental Material 1**(.pdf)

Uncropped western blot image for Figure 1**A** and **C**, Figure 2**B**, Figure 4**A**, Figure 6**B**, Figure 7**A** and **D**.

2- File Name: **Supplemental Material 2**(.docx)

Supplementary Table 1 - Clinical data of non-diabetic (N) and diabetic mice (D)

Supplementary Table 2 – Basic characteristics of human RPE cadaver tissue donors
