# Supplementary material for: Aberrant Akt2 signaling in the RPE may contribute to retinal fibrosis process in diabetic retinopathy
Source: Cell Death Discov. 2023 Jul 13;9:243. doi: 10.1038/s41420-023-01545-4 (PMC10345150; doi:10.1038/s41420-023-01545-4)
Supplement: Supplementary file 2 — Supplementary Table [file 41420_2023_1545_MOESM2_ESM.docx]

**Supplementary Table 1 - Clinical data of non-diabetic (N) and diabetic mice (D)**

| Group | Fasted blood glucose (mg/dL) | Body weight (g) | HbA_1c_ | |
| --- | --- | --- | --- | --- |
|  |  |  | % | mmol/mol |
| WT-N | 95 ± 15 | 43 ± 5 | 3.0 ± 0.1 | 9.7 ± 0.9 |
| WT-D | 464 ± 32 | 30 ± 1***** | 8.3 ± 0.3***** | 67 ± 3.3***** |
| *Akt2*^fl/fl^ -N | 103 ± 13 | 41 ± 5 | 3.0 ± 0.1 | 9.5 ± 0.5 |
| *Akt2*^fl/fl^ -D | 466 ± 23***** | 30 ± 2***** | 8.01± 0.3***** | 65 ± 2.6***** |
| *Akt2* cKO-N | 102 ± 17 | 41 ± 4 | 3.1 ± 0.1 | 9.6 ± 0.7 |
| *Akt2* cKO-D | 472 ± 29***** | 30 ± 2***** | 8.2 ± 0.2***** | 66 ± 2.4***** |

**Supplementary Table 2 – Basic characteristics of human RPE cadaver tissue donors**

| Group | Age (years)  Average | Male  (Number) | Female  (Number) | Duration of Diabetes (Years) | HbA1c (%) | Race |
| --- | --- | --- | --- | --- | --- | --- |
| ND | 70 ± 8 | 4 | 2 | - | - | Caucasian |
| DR | 72 ± 9 | 4 | 2 | 10 ± 6.5 | 7.2 + 1.6 | Caucasian |
