# Supplementary material for: Aberrant Akt2 signaling in the RPE may contribute to retinal fibrosis process in diabetic retinopathy
Source: Cell Death Discov. 2023 Jul 13;9:243. doi: 10.1038/s41420-023-01545-4 (PMC10345150; doi:10.1038/s41420-023-01545-4)
Supplement: Supplementary file 3 — Supplementary Table Legends [file 41420_2023_1545_MOESM3_ESM.docx]

**Table legends**

**Supplementary Table 1 - Clinical data of non-diabetic (N) and diabetic mice (D)**

Data are mean ± SD.  In short term studies, *n* = 18 for WT-N, *n* = 42 for WT-D, *n* = 25 for *Akt2*^fl/fl^-N, *n* = 27 for *Akt2*^fl/fl^-D, *n* = 20 *Akt2* cKO-N and *Akt2* cKO-D. **p* < 0.0001 compared with appropriate N from each group. Statistical test used in this study is Two-tailed, Mann-Whitney *U*-test. N, Non-diabetic; D, Diabetic; WT, Wildtype; cKO, conditional knock-out.

**Supplementary Table 2 – Basic characteristics of human RPE cadaver tissue donors**

Data are mean ± SD. *n* = 6 for each group, Duration of Diabetes and HbA1c data is not accessible for one female case in DR group. ND, Non-diabetic; DR, Diabetic retinopathy.
