# Supplementary material for: Aberrant Akt2 signaling in the RPE may contribute to retinal fibrosis process in diabetic retinopathy
Source: Cell Death Discov. 2023 Jul 13;9:243. doi: 10.1038/s41420-023-01545-4 (PMC10345150; doi:10.1038/s41420-023-01545-4)
Supplement: Supplementary file 4 — Original Data File [file 41420_2023_1545_MOESM4_ESM.pdf]

Figure 1 A

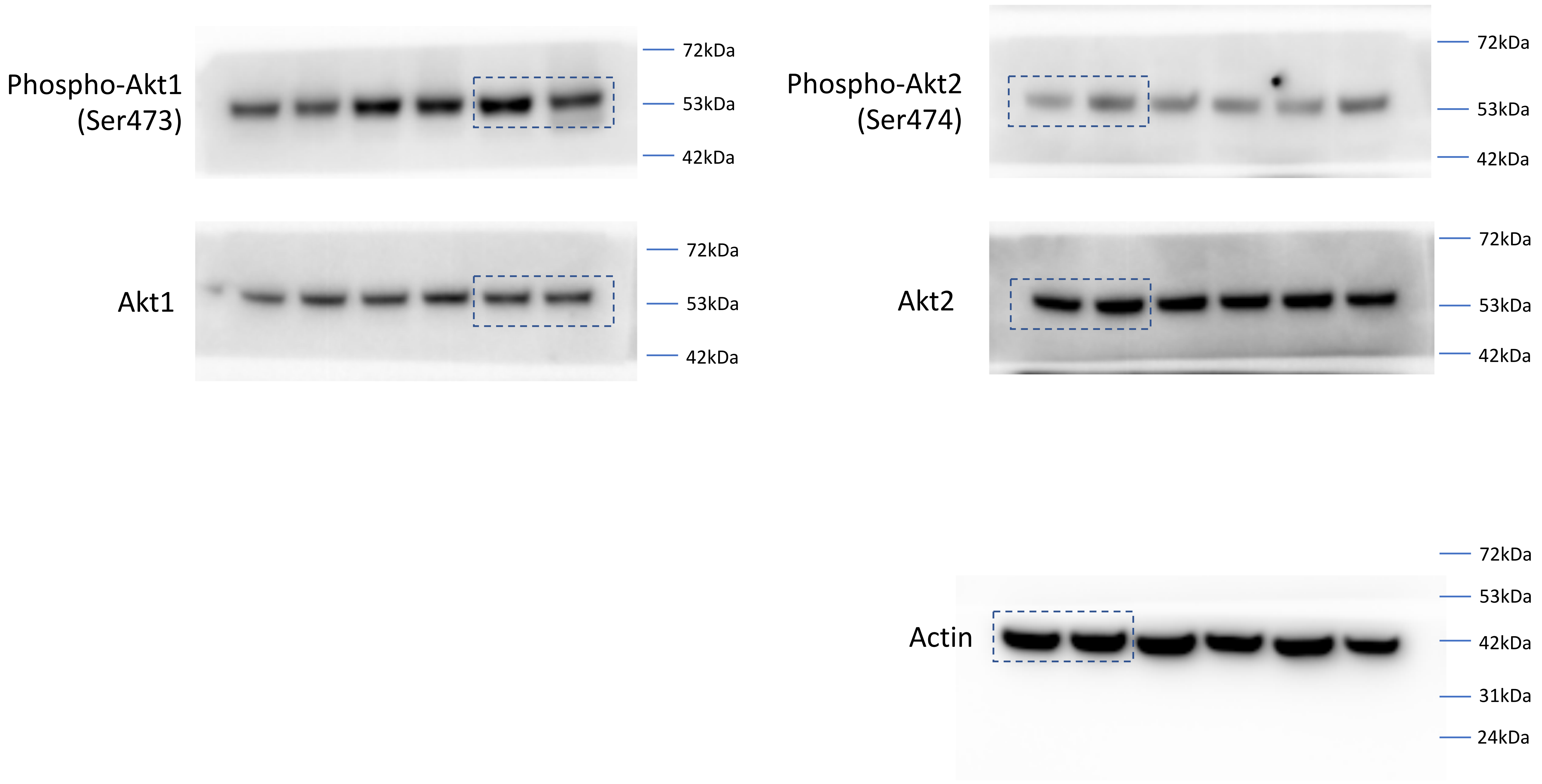
Phospho-Akt1
(Ser473)
72kDa
53kDa
42kDa
Akt1
72kDa
53kDa
42kDa
Phospho-Akt2
(Ser474)
72kDa
53kDa
42kDa
Akt2
72kDa
53kDa
42kDa
Actin
72kDa
53kDa
42kDa
31kDa
24kDa

Figure 1 C

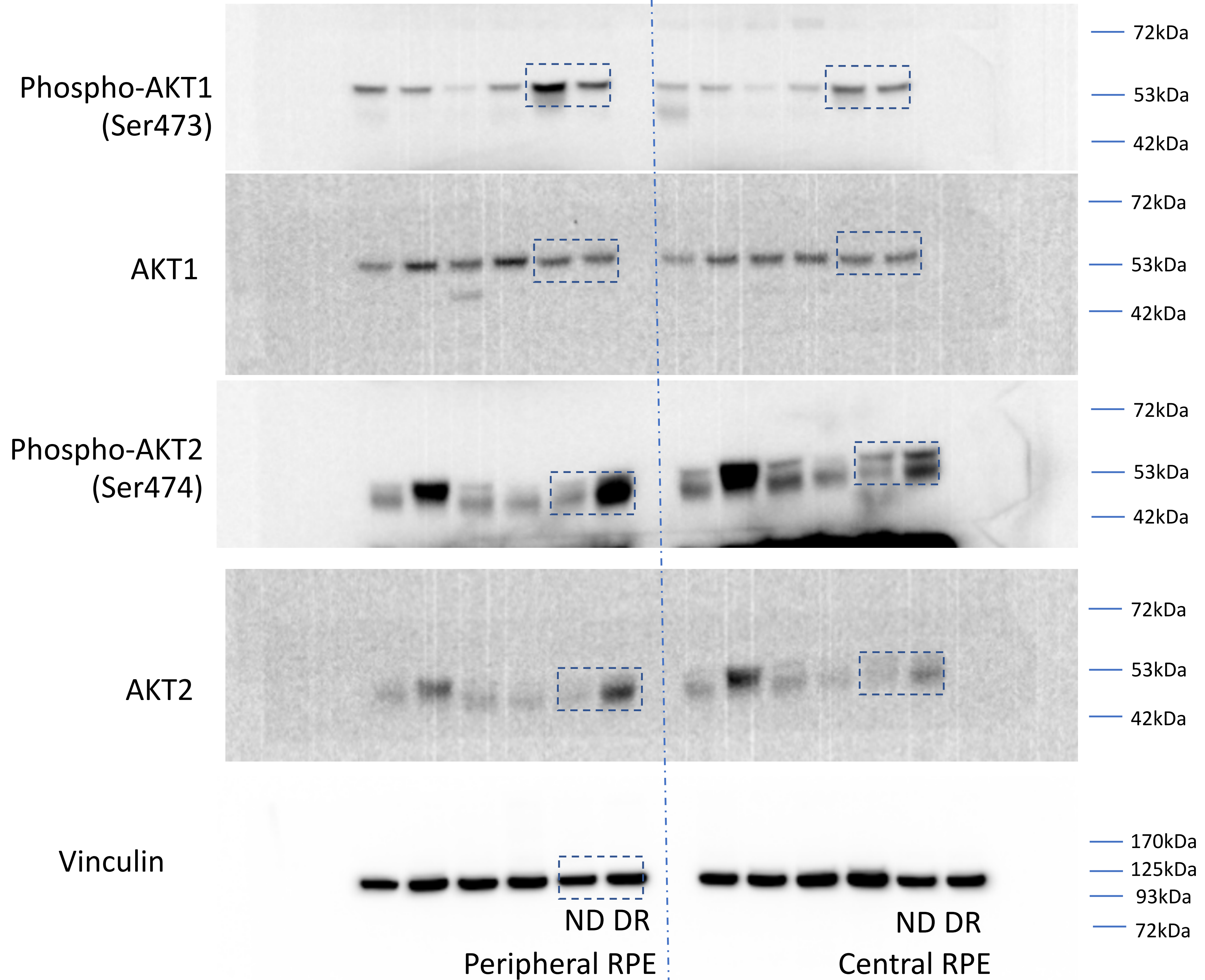

Phospho-AKT1 (Ser473)
AKT1
Phospho-AKT2 (Ser474)
AKT2
Vinculin
72kDa
53kDa
42kDa
72kDa
53kDa
42kDa
72kDa
53kDa
42kDa
72kDa
53kDa
42kDa
170kDa
125kDa
93kDa
72kDa
ND DR
Peripheral RPE
ND DR
Central RPE

Figure 2 B

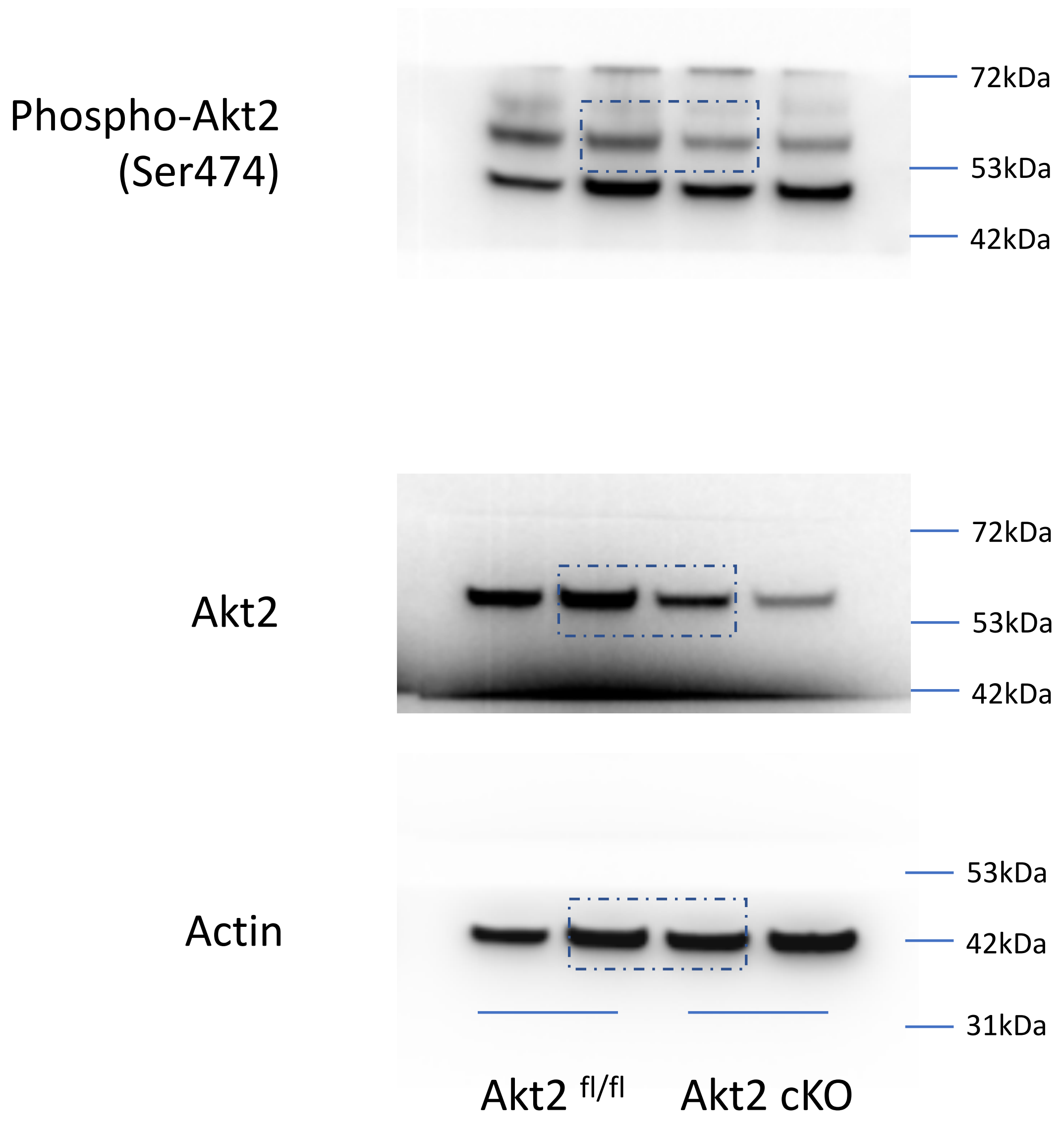
Phospho-Akt2
(Ser474)
72kDa
53kDa
42kDa
Akt2
72kDa
53kDa
42kDa
Actin
53kDa
42kDa
31kDa
Akt2 fl/fl
Akt2 cKO

Figure 4 A

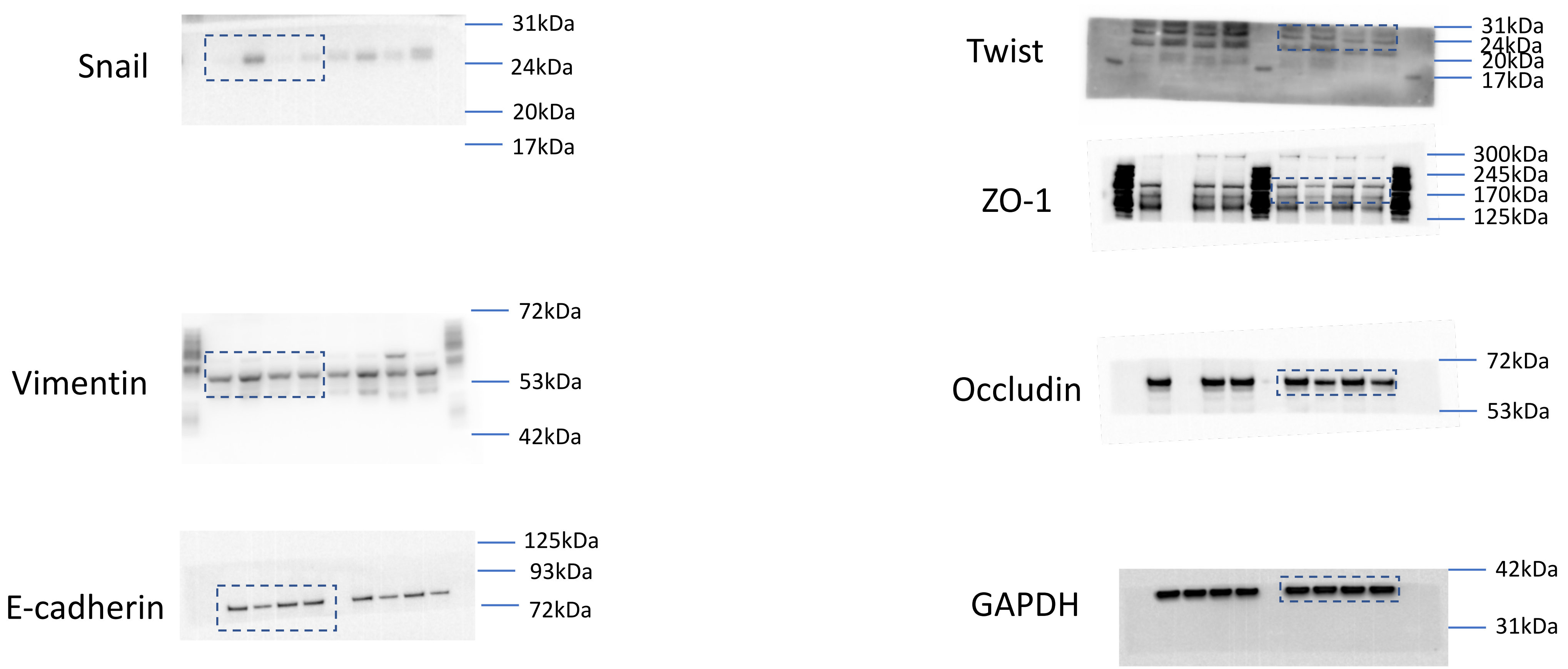
Snail
31kDa
24kDa
20kDa
17kDa
Vimentin
72kDa
53kDa
42kDa
E-cadherin
125kDa
93kDa
72kDa
Twist
31kDa
24kDa
20kDa
17kDa
ZO-1
300kDa
245kDa
170kDa
125kDa
Occludin
72kDa
53kDa
GAPDH
42kDa
31kDa

Figure 6 B

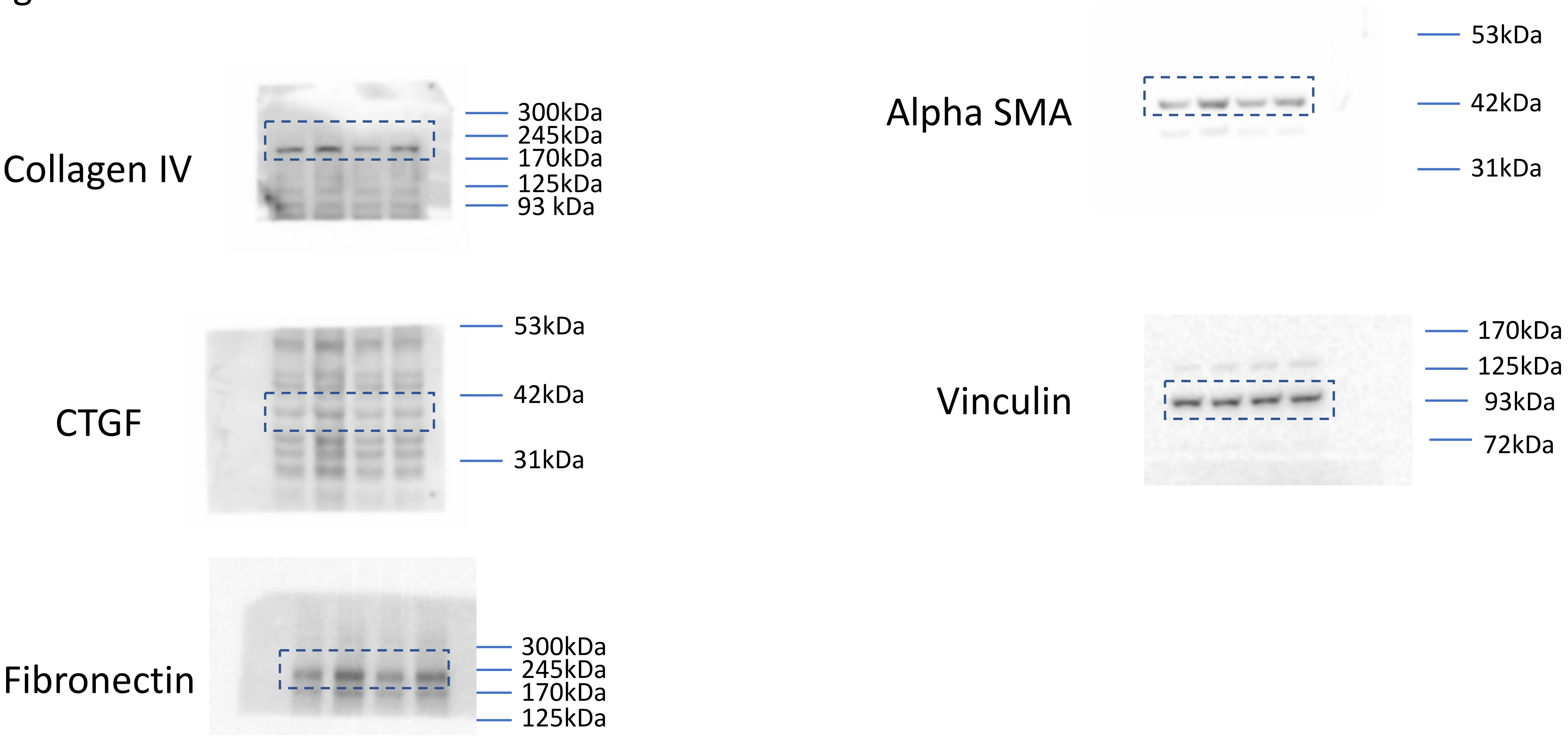
Collagen IV
300kDa
245kDa
170kDa
125kDa
93 kDa
CTGF
53kDa
42kDa
31kDa
Fibronectin
300kDa
245kDa
170kDa
125kDa
Alpha SMA
53kDa
42kDa
31kDa
Vinculin
170kDa
125kDa
93kDa
72kDa

Figure 7 A

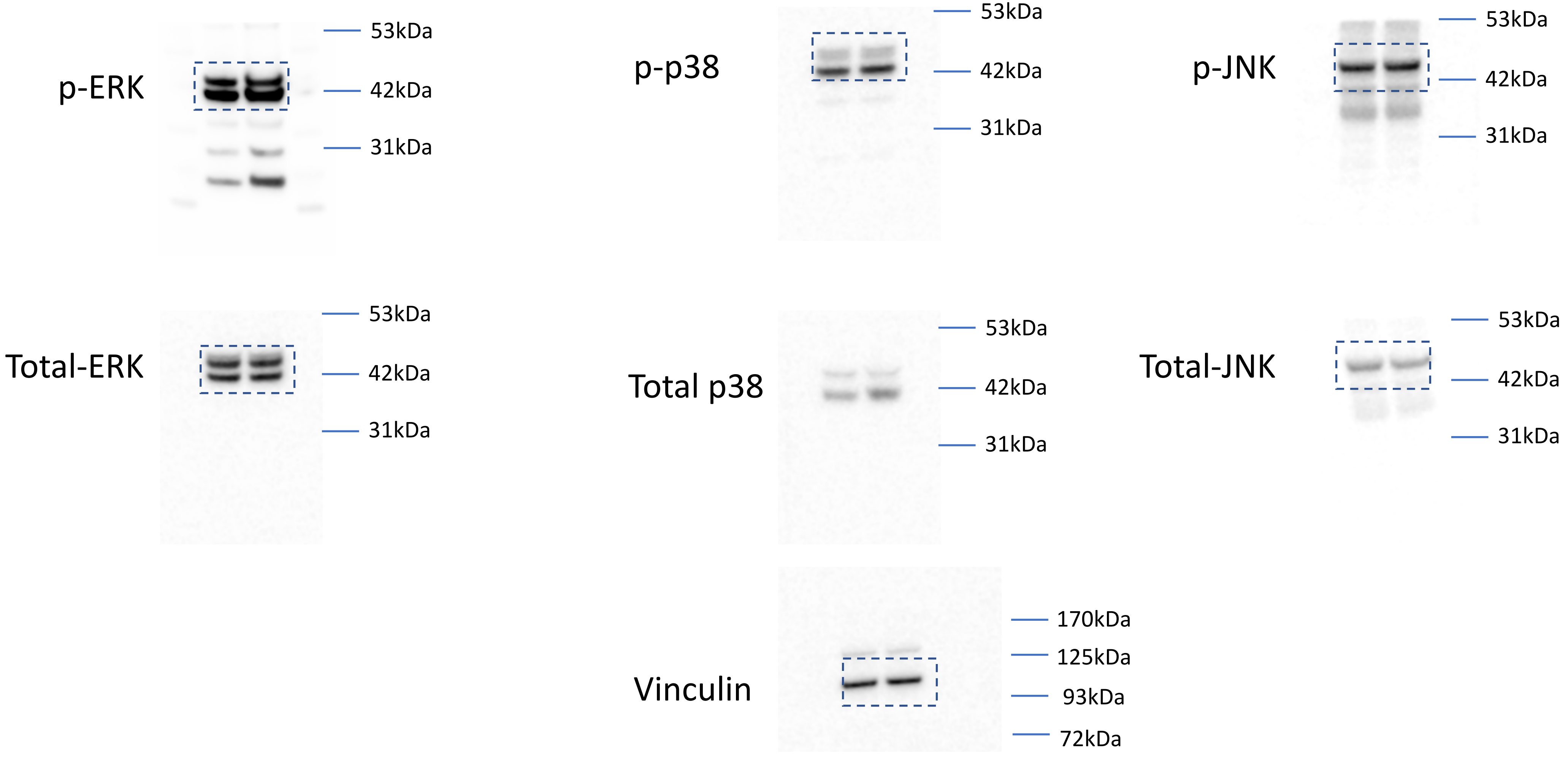
p-ERK
53kDa
42kDa
31kDa
p-p38
53kDa
42kDa
31kDa
p-JNK
53kDa
42kDa
31kDa
Total-ERK
53kDa
42kDa
31kDa
Total p38
53kDa
42kDa
31kDa
Total-JNK
53kDa
42kDa
31kDa
Vinculin
170kDa
125kDa
93kDa
72kDa

Figure 7 D

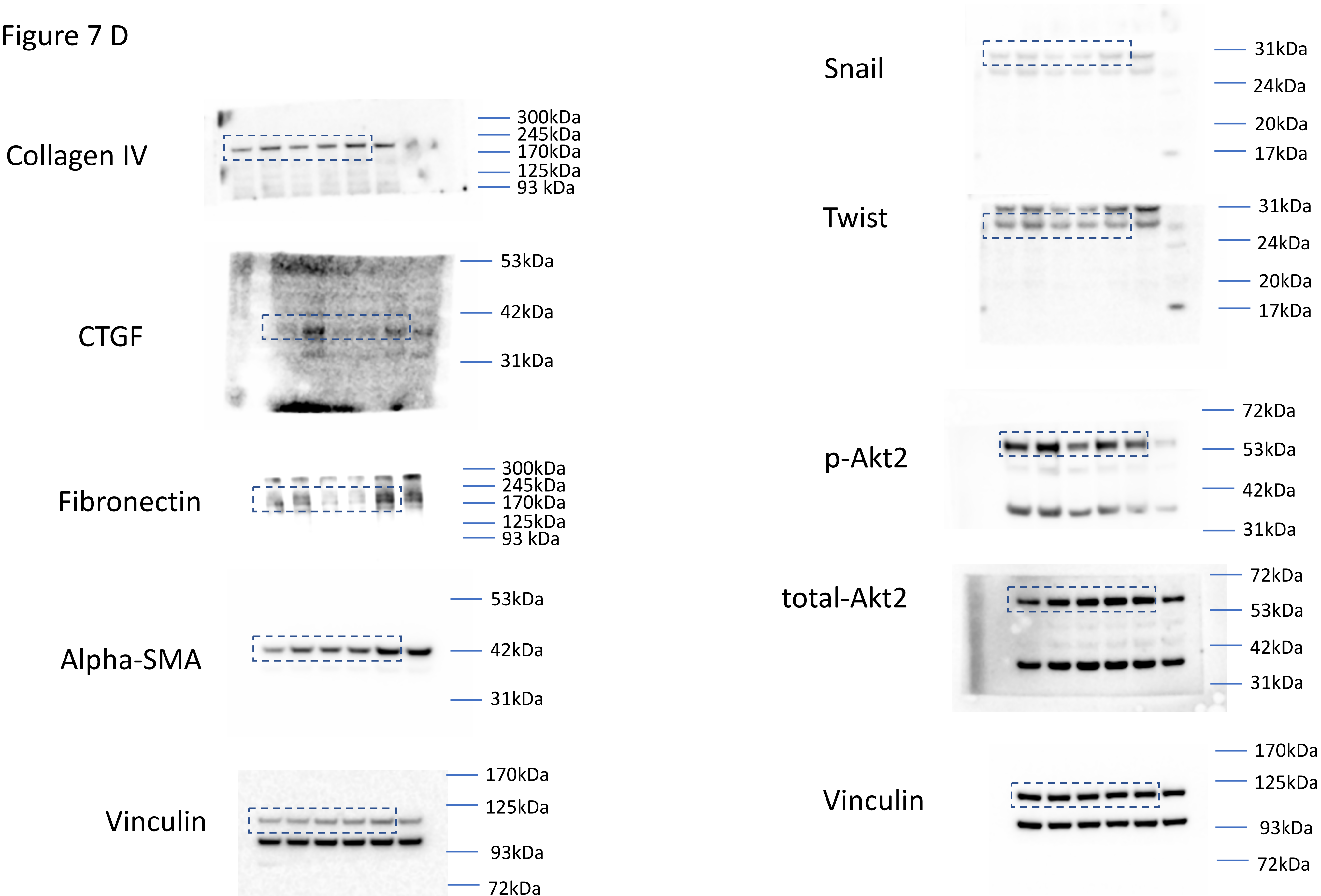
Collagen IV
300kDa
245kDa
170kDa
125kDa
93 kDa
CTGF
53kDa
42kDa
31kDa
Fibronectin
300kDa
245kDa
170kDa
125kDa
93 kDa
Alpha-SMA
53kDa
42kDa
31kDa
Vinculin
170kDa
125kDa
93kDa
72kDa
Snail
31kDa
24kDa
20kDa
17kDa
Twist
31kDa
24kDa
20kDa
17kDa
p-Akt2
72kDa
53kDa
42kDa
31kDa
total-Akt2
72kDa
53kDa
42kDa
31kDa
Vinculin
170kDa
125kDa
93kDa
72kDa
